# Agreement between upper-arm and wrist cuff occlusion for the assessment of vascular reactivity by digital thermal monitoring

**DOI:** 10.3389/fphys.2026.1961953

**Published:** 2026-10-12

**Authors:** Hamidreza Mohammadi, Ali Hashemi, Timothy O’Brien, Hamed Ghoshouni, Hirofumi Tanaka, Robert A. Kloner, Morteza Naghavi

**Affiliations:** 1 American Heart Technologies, Houston, TX, United States; 2 Temperature Specialists Inc., St. Francis, MN, United States; 3 The University of Texas at Austin, Austin, TX, United States; 4 Huntington Medical Research Institutes, Pasadena, CA, United States; 5 Keck School of Medicine of University of Southern California, Los Angeles, CA, United States

**Keywords:** agreement, digital thermal monitoring, endothelial function, method-comparison, reactive hyperemia, vascular reactivity index

## Abstract

**Background:**

Digital Thermal Monitoring (DTM) is an automated method for assessing vascular reactivity using the vascular reactivity index (VRI), a temperature-derived surrogate marker associated with endothelial function. Standard DTM requires five minutes of supra-systolic upper-arm cuff occlusion, which may cause discomfort and limit feasibility in some individuals. Whether wrist cuff occlusion provides comparable VRI measurements remains unknown.

**Methods:**

In this method-comparison study, 48 healthy adults underwent DTM assessment using both upper-arm and wrist cuff occlusion in randomized order during the same visit. Agreement between the two occlusion sites for VRI, pulse rate (PR), and blood pressure (BP) was evaluated using paired comparisons and agreement analyses.

**Results:**

Mean VRI was 1.70 ± 0.33 with upper-arm cuff occlusion and 1.76 ± 0.34 with wrist cuff occlusion, with no significant difference between sites (mean difference 0.06, 95% CI −0.04 to 0.15; p=0.25) and no significant proportional bias. Agreement for VRI was moderate (ICC = 0.53, 95% CI 0.29–0.70). In contrast, wrist measurements overestimated systolic BP by 10.5 mmHg (95% CI 7.3–13.7; p<0.001) and diastolic BP by 10 mmHg (95% CI 7.1–12.9; p<0.001), with poor agreement (ICC = 0.29, 95% CI −0.05 to 0.56 and ICC = 0.18, 95% CI −0.07 to 0.43, respectively).

**Conclusions:**

Wrist cuff occlusion provides similar mean VRI values to upper-arm occlusion but demonstrates only moderate agreement at the individual level. Further studies with larger and more diverse populations, including repeated measurements, are required assessment before wrist and upper-arm cuff occlusion can be considered interchangeable for individual assessment.

## Introduction

The vascular endothelium plays a central role in maintaining vascular homeostasis by regulating vascular tone, inflammation, thrombosis, and tissue perfusion (Segal, 2005; Daiber et al., 2017; Dri et al., 2023). In response to increased shear stress, endothelial cells release nitric oxide (NO) and other vasodilatory mediators that promote arterial relaxation. Impairment of this response represents one of the earliest manifestations of endothelial dysfunction and atherosclerosis (Libby et al., 2002; Hsieh et al., 2014; Xu et al., 2021). Because endothelial dysfunction precedes overt cardiovascular disease and is associated with adverse cardiovascular outcomes (Hadi et al., 2005), assessment of vascular reactivity has become an important tool for identifying individuals at increased cardiovascular risk beyond conventional risk factors (Thijssen et al., 2019; Mućka et al., 2022).

Endothelial function can be assessed non-invasively using several techniques. Flow-Mediated Dilation (FMD) of the brachial artery has been the most popular non-invasive methodology used to assess endothelial function (Charakida et al., 2010; Thijssen et al., 2019; Mućka et al., 2022). Despite its utility, FMD is often impractical for routine clinical settings due to its reliance on expensive ultrasound equipment, high inter-observer variability, and the requirement for highly skilled technicians. As an alternative, Digital Thermal Monitoring (DTM) has emerged as a simplified, automated technique for assessing vascular reactivity. DTM-derived VRI has been shown to correlate with FMD, supporting its use as a surrogate marker of vascular function (Dhindsa et al., 2008).

This method measures fingertip temperature changes during a five-minute period of upper-arm cuff-induced reactive hyperemia to generate a Vascular Reactivity Index (VRI). VRI has been shown to correlate with established markers of atherosclerosis, such as coronary artery calcium scores, and serves as a surrogate measure of microvascular vascular reactivity (Ahmadi et al., 2008; Ahmadi et al., 2009; Naghavi et al., 2022).

A significant barrier to the widespread use of vascular reactivity tests is the patient discomfort associated with the standard protocol. The prolonged supra-systolic upper-arm occlusion required by the standard protocol may reduce patient tolerability in some individuals. Whether discomfort associated with cuff occlusion or differences in occlusion site influence the temperature-derived VRI measurement remains uncertain. A previous study found that placing the occlusion cuff at the base of the finger yielded mean VRI values comparable to those obtained using the conventional upper-arm protocol, although agreement between the two methods at the individual level was limited. These findings suggest that alternative sites of occlusion may be feasible for VRI assessment (Heath et al., 2021). A wrist-based occlusion protocol represents another potentially attractive alternative because it may improve patient comfort while remaining technically easier to perform in selected populations (Palatini et al., 2004; Komori et al., 2013). However, evidence from blood pressure (BP) measurement indicates that cuff location is not necessarily interchangeable. Wrist measurements frequently differ from brachial measurements because of hydrostatic influences, anatomical differences, and site-specific arterial characteristics (Palatini et al., 2004; Mahapatra et al., 2026). Whether a temperature-derived index of vascular reactivity such as VRI is similarly influenced by cuff location remains unknown. Therefore, we conducted a method-comparison study with the primary objective of evaluating the agreement between VRI measurements obtained using upper-arm and wrist cuff occlusion. As a secondary objective, we also compared these parameters between the two occlusion sites to determine whether any observed differences in VRI should be interpreted in the context of physiological variables known to exhibit varying degrees of site dependence.

## Methods

### Study design and participants

This was a single center, cross-sectional method-comparison study. Fifty healthy young adult volunteers were enrolled. Each participant underwent VRI assessment at two occlusion sites, the upper-arm and the wrist, during the same visit and thus served as their own control. Participants were excluded if they were pregnant, had undergone surgery or another medical intervention within the preceding 48 hours, or had a baseline fingertip temperature below 27 °C, as measurements obtained below this threshold are considered unreliable. Two participants met the temperature-based exclusion criterion, leaving 48 participants for the final analysis. No *a priori* sample-size calculation was performed, as this was an exploratory method-comparison study based on a convenience sample. With the achieved sample of 48 participants, each 95% limit of agreement for VRI was estimated with a precision of approximately ±0.16 units, which we considered adequate for this preliminary comparison. This study was conducted in accordance with the Declaration of Helsinki and was approved by the Institutional Review Board of the University of Houston (STUDY00000981). Written informed consent was obtained from all participants prior to enrollment.

### Study procedures

All assessments were performed in the morning after an overnight fast of at least 8 hours and abstinence from alcohol, tobacco, caffeine, vasoactive medication, and exercise. Measurements were conducted in a quiet room with controlled ambient temperature. Participants were seated for 15 minutes of rest before testing. Fingertip temperature was assessed before initiating the DTM procedure, and participants with a fingertip temperature below 27 °C were gently warmed during the resting period using an electric warming blanket placed over the shoulders and neck until the fingertip temperature reached ≥27 °C. Participants were randomly allocated to one of two sequence groups to control for order effects, using a computer-generated random allocation sequence. In Group 1, VRI was measured first with the occluding cuff on the upper-arm, and after a 30-minute rest, again with the cuff on the wrist; in Group 2 the order was reversed. The 30-minute interval was used to reduce potential carry-over between occlusions. During this interval, participants remained seated under the same environmental conditions, and fingertip temperature was reassessed before the second measurement. If fingertip temperature decreased below 27 °C, an electric warming blanket was applied over the shoulders and neck until the required temperature threshold was restored before initiating the second DTM assessment. In total, 23 participants were allocated to Group 1 and 25 to Group 2 (**Figure 1**).

**Figure 1:**
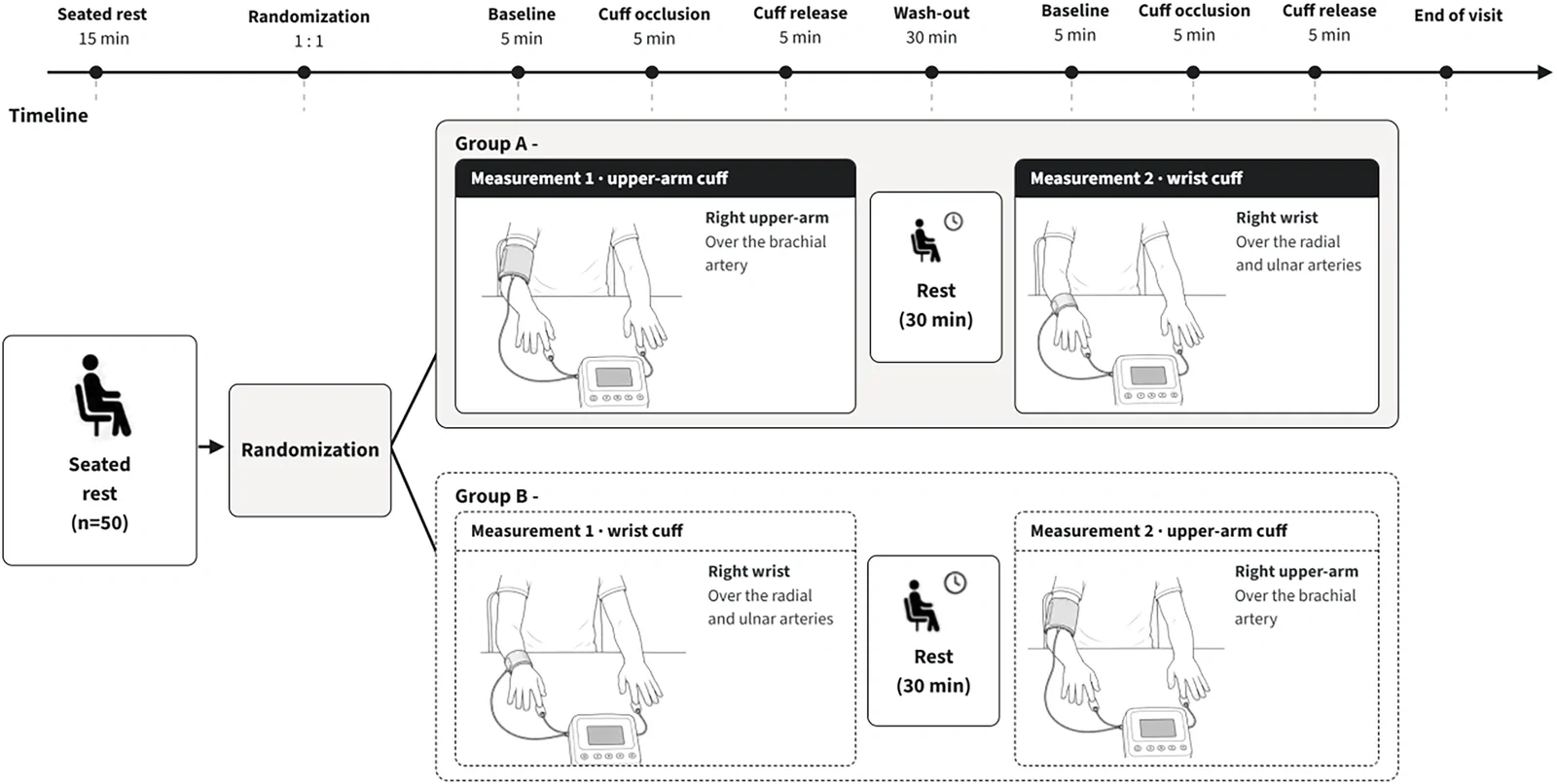
Randomized crossover study protocol. Participants were randomized 1:1 to undergo DTM assessment with upper-arm and wrist cuff occlusion in opposite sequences. All measurements were performed on the right side, with a 30-min wash-out period between measurements. The timing of baseline, cuff occlusion, and cuff release phases is shown in the timeline.

### VRI measurement

All participants underwent microvascular function assessment using the VENDYS-II device (model JK-001V, software version 1.2.4.102523.1036; Endothelix Inc., a subsidiary of American Heart Technologies, Houston, TX, USA), an automated office-based system. The VENDYS device uses DTM to capture microvascular responses to transient arterial occlusion and reperfusion. The procedure for measuring microvascular function using DTM has been described previously in detail (McQuilkin et al., 2009; Akhtar et al., 2010; Ley et al., 2011). At the beginning, skin-temperature sensors were affixed to the pads of both index fingers, and the cuff was applied at the site under study.

For upper-arm cuff occlusion, a medium-sized adult blood-pressure cuff (appropriate for an arm circumference of 27–35 cm) was positioned on the right upper-arm over the brachial artery, with its lower edge approximately 2–3 cm above the antecubital fossa. For wrist cuff occlusion, an appropriately sized wrist cuff was wrapped snugly around the right wrist, immediately proximal to the styloid processes, with the bladder centered over the volar aspect overlying the radial and ulnar arteries. In both configurations, cuff size was selected to match the circumference of the occluded segment. Throughout each measurement, participants remained seated, with both hands supported at the level of the heart to avoid hydrostatic effects on the cuff-derived pressure and on the occlusion stimulus.

Baseline blood pressure and fingertip temperatures were recorded during a 5-minute baseline period. For each protocol, the cuff was then inflated to 50 mmHg above the systolic BP measured at the corresponding cuff site (mean inflation pressure approximately 162 mmHg for upper-arm and 172 mmHg for wrist occlusion) and maintained for 5 minutes to induce transient ischemia. Complete arterial occlusion was confirmed by the device’s automated Incomplete Occlusion Flag; no incomplete occlusion events were detected in any measurement performed at either site. During this occlusion phase, fingertip temperature declines due to cessation of warm circulating blood. Upon cuff deflation after 5 minutes, reactive hyperemia ensues: blood flow rapidly returns and increases to the forearm and hand, resulting in a temperature rebound at the fingertip that is reflecting the magnitude of the post-ischemic microvascular reactive hyperemia response. The device continuously monitors temperature changes. Proprietary VENDYS software then calculates the VRI by quantifying the maximum difference between the observed temperature rebound curve and a theoretical zero-reactivity curve (ZRC) during the reactive hyperemia period, adjusted for starting fingertip temperature and ambient temperature. The ZRC acts as an internal control, exhibiting the anticipated temperature rebound when zero vascular reactivity is present and all other variables remain constant. VRI values generally range from 0.0 to 3.5 (Akhtar et al., 2010). The identical protocol was applied at both sites.

At the start of each test, the device automatically measured BP and PR from the occluding cuff, yielding systolic and diastolic BP and PR at both the upper-arm and wrist positions.

### Statistical analysis

All analyses were performed in Python 3.12 using the pandas and NumPy libraries for data handling, SciPy and stats models for statistical testing, and Matplotlib for figure generation. Continuous variables were presented as mean ± standard deviation (SD), and categorical variables as frequency and percentage. The mean difference between the two occlusion sites was tested against zero with a paired t-test. The correlations between the two sites of occlusion were evaluated by the Pearson correlation coefficient. Correlation was reported descriptively only, as it does not assess agreement. The Bland–Altman plots were used to assess agreement between upper-arm and wrist cuff occlusion for VRI, PR, systolic and diastolic BP (Bland and Altman, 1986). On each plot, the solid red line denotes the mean bias and the dashed lines denote the 95% limits of agreement (mean bias ± 1.96 × SD). The slope of the difference regressed on the mean was examined to check for proportional bias. Reliability was quantified with the intraclass correlation coefficient (ICC) computed as a two-way random-effects, absolute-agreement, single-measures model, ICC being interpreted as poor (<0.50), moderate (0.50–0.75), good (0.75–0.90) or excellent (>0.90) (Bland and Altman, 1986). A two-way model with site treated as a fixed factor was used, because the upper-arm and wrist are two specific, prespecified sites rather than raters sampled at random; for the absolute-agreement, single-measures form, the estimate is identical under the random- and mixed-effects specifications. Each ICC is reported with its 95% confidence interval.

Exploratory multivariable linear regression was used to examine whether the between-site difference in each measure was associated with participant characteristics (age, sex, and ethnicity). A two-sided p-value<0.05 was considered statistically significant.

## Results

A total of 48 participants were analyzed (mean age: 27.9 ± 2.1 years). Overall, 29 (60%) participants were male, and 13 (27%) were Hispanic or Latino. Participants were randomly assigned to the measurement sequence, with 23 undergoing upper-arm cuff occlusion and 25 undergoing wrist cuff occlusion. Fingertip and room temperatures at each protocol time point are presented in **Supplementary Tables S1 and S2**. Baseline fingertip temperatures were similar between the two conditions, and fingertip temperature declined during occlusion and recovered after cuff release in both conditions.

The wrist − arm VRI difference did not differ significantly between these two sequences (0.01 ± 0.40 vs 0.10 ± 0.24; p=0.34). For VRI, the mean was 1.70 ± 0.33 units with upper-arm cuff occlusion and 1.76 ± 0.34 units with wrist cuff occlusion; the mean difference (wrist − arm) was not significantly different from zero (bias 0.06 units, 95% CI −0.04 to 0.15; p=0.25), with 95% limits of agreement of −0.59 to 0.7 units. PR likewise did not differ significantly between the two sites (bias 0.3 bpm, 95% CI −2.1 to 2.8; p=0.78; limits of agreement −16.0 to 16.67 bpm). For both blood-pressure measures, the mean differed significantly between the two sites: systolic BP was higher at the wrist than the upper-arm, with a bias of 10.5 mmHg (95% CI 7.3 to 13.7; p<0.001) and limits of agreement of −11.2 to 32.1 mmHg, as was diastolic BP, with a bias of 10 mmHg (95% CI 7.1 to 12.9; p < 0.001) and limits of agreement of −9.70 to 29.70 mmHg. **Table 1** and **Figure 2** summarize the agreement between upper-arm and wrist cuff occlusion for all four measures. No statistically significant evidence of proportional bias was detected for any measure (all p ≥ 0.05; **Supplementary Table S3**).

**Table 1:** Comparison of VRI, pulse rate, and blood pressure between upper-arm and wrist cuff occlusion.

Measure	Upper-arm	Wrist	Bias (95% CI)	P-value *	95% LoA (95% CI) †
PR (bpm)	72.0 ± 9.6	72.3 ± 9.9	0.3 (−2.1 to 2.8)	0.78	−16.0 (−20.2 to −11.8) to 16.7 (12.5 to 20.9)
VRI (units)	1.70 ± 0.33	1.76 ± 0.34	0.06 (−0.04 to 0.15)	0.25	−0.59 (−0.75 to −0.42) to 0.70 (0.53 to 0.86)
Systolic BP (mmHg)	111.7 ± 10.6	122.1 ± 10.1	10.5 (7.3 to 13.7)	<0.001	−11.2 (−16.7 to −5.6) to 32.1 (26.6 to 37.7)
Diastolic BP (mmHg)	67.4 ± 8.5	77.4 ± 8.5	10 (7.1 to 12.9)	<0.001	−9.7 (−14.8 to −4.7) to 29.7 (24.7 to 34.8)

Values are presented as mean ± SD. Abbreviations: PR, pulse rate; VRI, vascular reactivity index; CI, confidence interval. *P values were calculated using the paired t-test. ^†^Lower and upper 95% limits of agreement.

**Figure 2:**
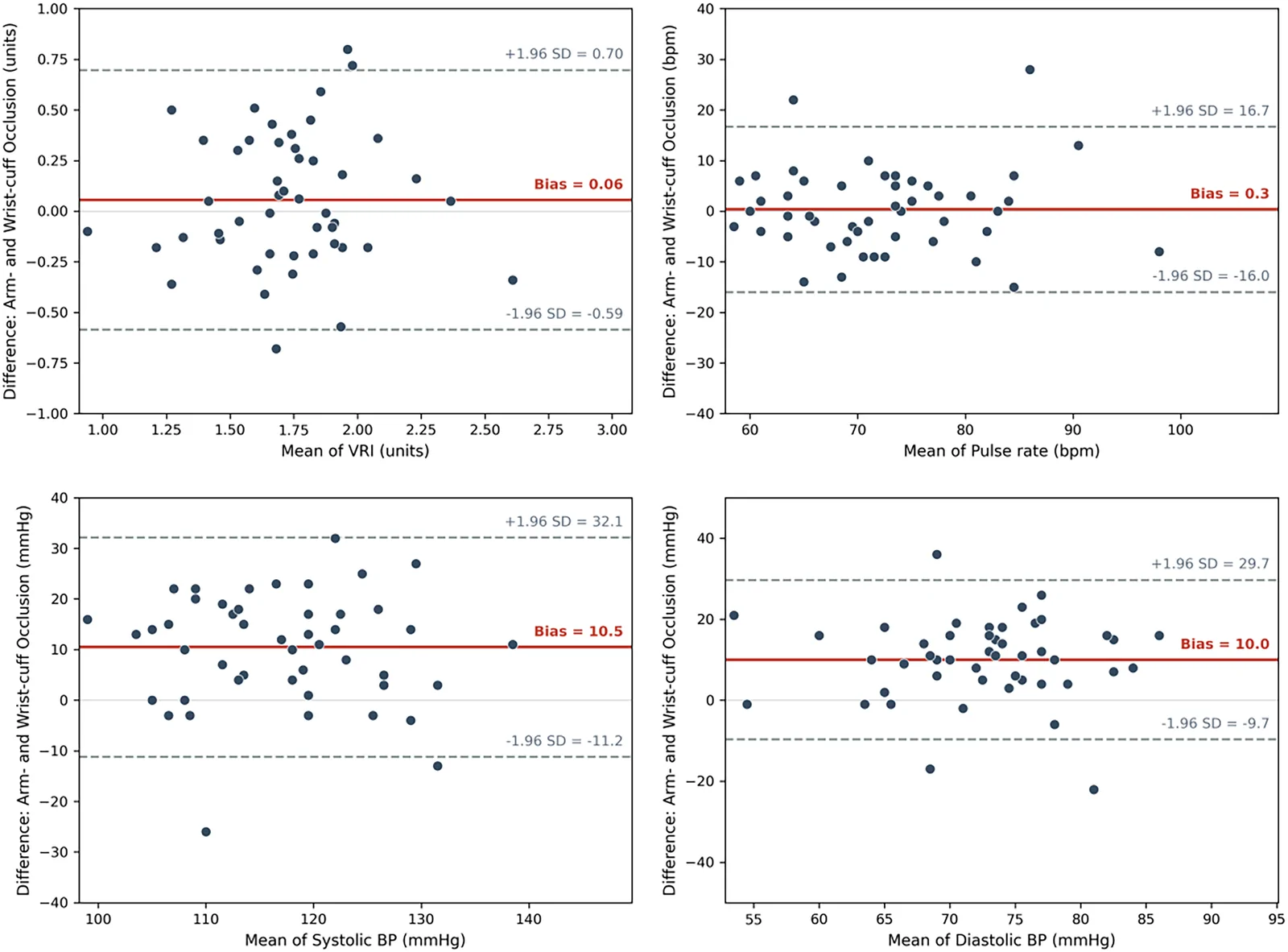
Bland–Altman plots of the difference between upper-arm and wrist cuff occlusion (wrist − arm) against the mean of the two measurements, for VRI, PR, systolic BP, and diastolic BP. Solid line = mean bias; dashed lines = 95% limits of agreement.

The correlation between the two occlusion sites is shown in the scatterplots in **Figure 3**. The correlation between upper-arm and wrist cuff occlusion was r = 0.63 (p<0.001) for PR, r = 0.53 (p<0.001) for VRI, r = 0.43 (p=0.002) for systolic BP, and r = 0.30 (p=0.04) for diastolic BP.

**Figure 3:**
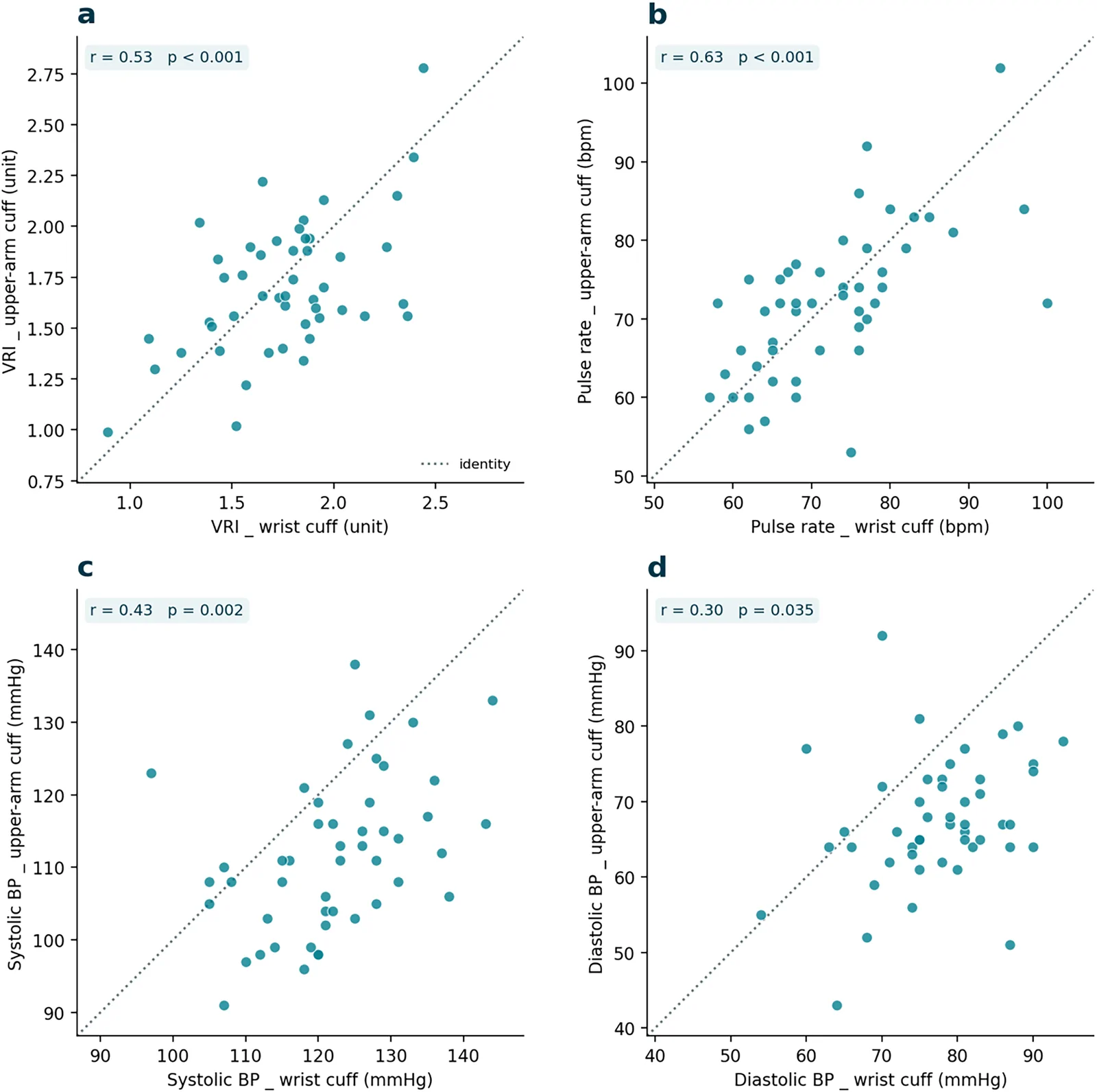
Scatterplots of VRI **(A)**, PR **(B)**, systolic BP **(C)**, and diastolic BP **(D)** measured with wrist cuff occlusion against upper-arm cuff occlusion. Each point represents one participant. The dashed line is the line of identity (y = x).

Reliability between the two occlusion sites, expressed as the absolute-agreement ICC, was moderate for PR (0.64, 95% CI 0.43 to 0.78) and VRI (0.53, 95% CI 0.29 to 0.70), and poor for systolic (0.29, 95% CI −0.05 to 0.56) and diastolic BP (0.18, 95% CI −0.07 to 0.43) (**Figure 4**).

**Figure 4:**
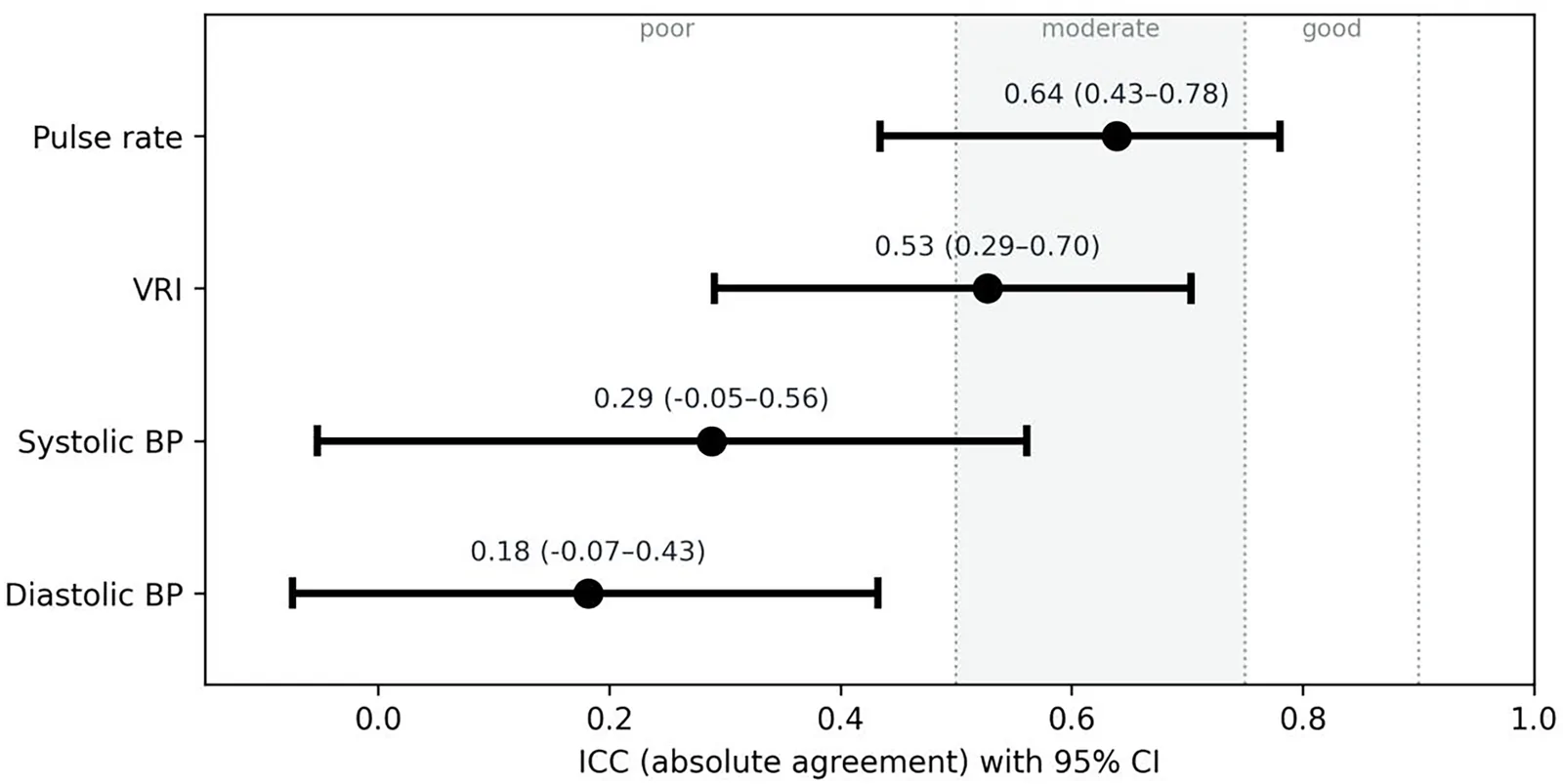
ICC (absolute agreement) with 95% CI for the agreement between upper-arm and wrist cuff occlusion, for PR, VRI, systolic BP, and diastolic BP. shaded band = moderate range (0.50–0.75).

In exploratory multivariable regression, the between-site difference (wrist − arm) was not associated with age, sex, or ethnicity for VRI, systolic BP, or diastolic BP (**Supplementary Table S4**).

## Discussion

VRI is a temperature-derived surrogate marker of vascular reactivity during reactive hyperemia. Although VRI has been associated with endothelial function-related measures, it does not directly quantify endothelial-dependent vasodilation. In this study, wrist cuff occlusion produced VRI values with no significant mean difference or proportional bias relative to the standard upper-arm protocol. However, absolute agreement was only moderate, and the observed limits of agreement indicate that the two approaches should not be assumed to be interchangeable for individual measurements. These findings underscore the distinction between similarity in group-level estimates and agreement at the participant level.

Our results are broadly consistent with those of Heath et al., who compared finger and brachial occlusion during DTM. Although mean VRI values did not differ between occlusion sites, the correlation was weak (Heath et al., 2021). The concordant pattern across the two studies suggests that changing the occlusion site may preserve the central tendency of VRI while introducing relevant within-person variability. Accordingly, evaluations of alternative DTM protocols should include measures of absolute agreement and limits of agreement rather than relying on mean differences or correlation alone.

Studies on the assessment of vascular endothelial function using FMD showed that the location of the cuff can have an impact on the findings of the FMD protocol. Berry et al. reported that upper-arm occlusion generated greater peak reactive hyperemia, a larger brachial artery FMD response, and a later peak dilation than forearm occlusion (Berry et al., 2000). Mannion et al. similarly observed greater hyperemic flow and FMD with cuff placement above rather than below the elbow, although proximal occlusion caused more discomfort (Mannion et al., 1998). Doshi et al. demonstrated that the FMD response induced by wrist occlusion was entirely dependent on NO, whereas upper-arm occlusion produced a vasodilatory response with a considerable NO-independent component, likely related to ischemia in the tissues surrounding the brachial artery. These findings suggest that cuff location and the volume of occluded tissue can influence post-ischemic vascular responses (Doshi et al., 2001); however, the mechanistic implications of NO dependence and endothelial specificity described in FMD cannot be directly extrapolated to VRI. In the present study, we therefore consider these findings only in relation to the broader physiological concept that occlusion site and the extent of ischemic tissue involvement may affect the characteristics of post-ischemic hyperemia, which may contribute to differences in fingertip temperature responses measured by VRI.

The parallel analyses of BP and PR provide useful internal context. PR, which should be comparatively insensitive to cuff location, showed the highest agreement between wrist and upper-arm measurements. In contrast, wrist BP values were approximately 10 mmHg higher for both systolic and diastolic pressure and demonstrated poor agreement with upper-arm measurements. This pattern is consistent with the recognized site dependence of wrist BP, including hydrostatic influences and differences in arterial geometry and stiffness (Palatini et al., 2004; Mahapatra et al., 2026) and confirms that the two cuff positions represent distinct hemodynamic measurement environments rather than simply interchangeable placements. The absence of a corresponding systematic difference in VRI is nevertheless noteworthy. Unlike cuff-derived BP, VRI is calculated from the fingertip temperature rebound after cuff release and is adjusted for baseline fingertip and ambient temperature relative to a modeled zero-reactivity curve. These features may reduce the extent to which site-dependent pressure differences translate into a consistent directional effect on VRI, and the similar magnitude of fingertip temperature decline during occlusion at both sites is consistent with a broadly comparable ischemic stimulus (Naghavi et al., 2016; Naghavi et al., 2022). The remaining disagreement may reflect a combination of differences in the ischemic vascular territory, local collateral and microvascular responses, and the inherent biological and measurement variability of reactive hyperemia. Because repeat measurements were not obtained at either site, the present study cannot separate true between-site variability from within-site test-retest variability.

These findings have practical implications. Wrist cuff occlusion may be useful in situations where upper-arm occlusion is technically challenging or where patient tolerability is a concern; however, the present study did not directly assess comfort or feasibility. Under standardized conditions, the absence of systematic mean bias supports further evaluation of the wrist protocol for group-level research applications. However, the present results do not establish equivalence, and the same occlusion site should be maintained when serial measurements are used to assess change within an individual.

The patient-centered rationale for wrist-based testing is plausible but remains indirect in the DTM setting. In the COOL-BP study, 91% of participants who used both a cuffless wrist monitor and an upper-arm home BP cuff preferred the wrist device, primarily because of ease of use and convenience (Unlu et al., 2025). Zeng et al. found that patients considered a wrist ambulatory BP monitor more comfortable than an arm-type device, although the wrist monitor had a lower successful-recording rate and substantial individual measurement discrepancies (Zeng et al., 2021). Kawaja et al. likewise showed that conventional arm-cuff ambulatory monitoring could be cumbersome, disruptive to daily activities, or embarrassing, while some users of wrist devices remained concerned about discomfort, movement-related error, and sensor displacement (Kawaja et al., 2025). Because these studies evaluated cuffless or ambulatory BP devices rather than a 5-minute supra-systolic DTM occlusion, their findings should not be directly extrapolated to the present protocol.

Taken together, wrist cuff occlusion appears promising as a technically convenient alternative for selected settings, but its role should be defined by the intended level of inference. Similar mean VRI values may be adequate for some cross-sectional group comparisons, whereas the moderate individual agreement argues against switching between wrist and upper-arm occlusion during longitudinal follow-up or using site-specific results interchangeably in clinical decision-making. Replication in larger and more diverse cohorts, together with repeated measurements at each site and prespecified acceptable limits of agreement, is required before broader adoption.

## Limitations

Several limitations should be considered. First, the sample size was modest and resulted in relatively wide confidence intervals for the ICC. Future studies should be powered specifically for agreement estimates. Second, the cohort consisted of young and healthy adults, limiting generalizability to older or higher-risk populations. Third, only a single measurement was obtained at each site; therefore, within-site test–retest repeatability could not be assessed. The observed limits of agreement may reflect biological variability, measurement error, and true cuff-location effects, which cannot be separated in the present design. No prespecified acceptable difference for VRI was defined, as no validated threshold for a meaningful VRI difference has been established. Therefore, this study evaluates agreement rather than equivalence or interchangeability. Finally, VRI should be interpreted as a temperature-derived surrogate marker of vascular reactivity rather than a direct measure of endothelial function. Discomfort, tolerability, preference, and technical feasibility were not assessed, and the potential practical advantages of wrist occlusion require further evaluation.

## Conclusions

VRI obtained using wrist cuff occlusion showed no systematic bias relative to the standard upper-arm cuff occlusion but demonstrated only moderate agreement at the individual level. In contrast, wrist BP measurements showed systematic overestimation and poor agreement with upper-arm measurements, whereas PR demonstrated the highest agreement between sites. Wrist cuff occlusion may therefore represent a practical alternative for group-level assessment of VRI. However, larger studies in more diverse populations that incorporate within-site repeated measurements, a predefined agreement margin, VRI-category concordance, and direct comfort/feasibility assessment are required before wrist occlusion can be adopted as an alternative to the standard protocol.

## Data Availability

The raw data supporting the conclusions of this article will be made available by the authors, without undue reservation.
