# Supplementary material for: Agreement between upper-arm and wrist cuff occlusion for the assessment of vascular reactivity by digital thermal monitoring
Source: Front Physiol. 2026 Oct 12;17:1961953. doi: 10.3389/fphys.2026.1961953 (PMC13696328; doi:10.3389/fphys.2026.1961953)
Supplement: Supplementary file 1 [file Table1.docx]

**Supplementary Material**

**Table S1.** Fingertip and room temperatures during upper-arm cuff occlusion.

|  | **1 s** | **300 s** | **600 s** | **900 s** |
| --- | --- | --- | --- | --- |
| Fingertip temperature, left (°C) | 31.95 ± 1.06 | 32.45 ± 1.09 | 32.47 ± 0.97 | 32.40 ± 1.14 |
| Fingertip temperature, right (°C) | 31.63 ± 1.09 | 32.29 ± 0.96 | 29.60 ± 1.22 | 32.15 ± 1.03 |
| Room temperature (°C) | 24.56 ± 1.90 | 24.54 ± 1.87 | 24.57 ± 1.86 | 24.59 ± 1.86 |

*Values are mean ± SD. The right hand was the occluded side; the left hand served as the contralateral control. Time points correspond to the start of the baseline period (1 s), the end of baseline (300 s), the occlusion phase (600 s), and the post-deflation reactive-hyperemia phase (900 s).*

**Table S2.** Fingertip and room temperatures during wrist cuff occlusion.

|  | **1 s** | **300 s** | **600 s** | **900 s** |
| --- | --- | --- | --- | --- |
| Fingertip temperature, left (°C) | 31.97 ± 1.10 | 32.42 ± 1.00 | 32.51 ± 1.01 | 32.51 ± 1.12 |
| Fingertip temperature, right (°C) | 31.66 ± 1.17 | 32.24 ± 1.06 | 29.67 ± 1.15 | 32.24 ± 1.02 |
| Room temperature (°C) | 24.55 ± 1.95 | 24.56 ± 1.93 | 24.56 ± 1.95 | 24.63 ± 1.99 |

*Values are mean ± SD. The right hand was the occluded side; the left hand served as the contralateral control. Time points as in Table S1. One participant's recording ended before 600 s, giving n = 48 at the 600 s and 900 s time points.*

**Table S3.** Assessment of proportional bias: regression of the between-site difference on the mean of the two measurements.

| **Measure** | **Slope (β)** | **95% CI** | **P-value** |
| --- | --- | --- | --- |
| PR (bpm) | 0.039 | −0.24 to 0.32 | 0.78 |
| VRI (units) | 0.048 | −0.28 to 0.38 | 0.77 |
| Systolic BP (mmHg) | −0.071 | −0.44 to 0.30 | 0.70 |
| Diastolic BP (mmHg) | −0.001 | −0.44 to 0.43 | 0.99 |

*Differences are calculated as wrist − arm. β, regression slope; CI, confidence interval. A slope not significantly different from zero indicates that the between-site difference was constant across the*

In multivariable linear regression, the between-site difference (wrist − arm) was not associated with age, sex, or ethnicity for VRI, systolic BP, or diastolic BP (all p> 0.05; model R² = 0.07, 0.06, and 0.05, respectively). For PR, the difference was greater in Hispanic than non-Hispanic participants (p = 0.02); given the multiple comparisons performed and the small size of this subgroup, this isolated association is likely a chance finding

**Table S4.** Exploratory multivariable linear regression of the between-site difference (wrist − arm) on participant characteristics.

| **Outcome (difference)** | **Predictor** | **β (95% CI)** | **p** | **Model R²** |
| --- | --- | --- | --- | --- |
| **VRI** | Age | −0.02 (−0.07 to 0.02) | 0.30 | 0.07 |
|  | Male sex (vs female) | −0.14 (−0.33 to 0.06) | 0.17 |  |
|  | Hispanic (vs non-Hispanic) | 0.02 (−0.20 to 0.23) | 0.87 |  |
| **Systolic BP** | Age | −0.67 (−2.19 to 0.86) | 0.38 | 0.06 |
|  | Male sex (vs female) | 3.03 (−3.59 to 9.65) | 0.36 |  |
|  | Hispanic (vs non-Hispanic) | −3.50 (−10.78 to 3.78) | 0.34 |  |
| **Diastolic BP** | Age | −0.80 (−2.20 to 0.60) | 0.25 | 0.05 |
|  | Male sex (vs female) | 1.81 (−4.26 to 7.87) | 0.55 |  |
|  | Hispanic (vs non-Hispanic) | −1.91 (−8.57 to 4.76) | 0.57 |  |
| **Pulse rate** | Age | −0.00 (−1.12 to 1.11) | 0.99 | 0.12 |
|  | Male sex (vs female) | −1.08 (−5.91 to 3.75) | 0.66 |  |
|  | Hispanic (vs non-Hispanic) | 6.20 (0.89 to 11.51) | 0.02 |  |
